# Fluorescent Nano-Biomass Dots: Ultrasonic-Assisted Extraction and Their Application as Nanoprobe for Fe^3+^ detection

**DOI:** 10.1186/s11671-019-2950-x

**Published:** 2019-04-15

**Authors:** Wen-Bo Zhao, Kai-Kai Liu, Shi-Yu Song, Rui Zhou, Chong-Xin Shan

**Affiliations:** Henan Key Laboratory of Diamond Optoelectronic Materials and Devices, School of Physics and Engineering, Zhengzhou University, No. 75 Daxue Road, Zhengzhou, 450052 People’s Republic of China

**Keywords:** Nano-biomass dots, Fluorescence, Ultrasonic methods, Nanoprobe, Fe^3+^ detection

## Abstract

Biomass as sustainable and renewable resource has been one of the important energy sources for human life. Herein, luminescent nano-biomass dots (NBDs) have been extracted from soybean through ultrasonic method, which endows biomass with fluorescence property. The as-prepared NBDs are amorphous in structure with an average diameter of 2.4 nm and show bright blue fluorescence with a quantum yield of 16.7%. Benefiting from the edible raw materials and heating-free synthesis process, the cytotoxicity test shows that the cell viability still keeps 100% even if the concentration of the NBDs reaches 800 μg/ml, indicating the good biocompatibility of the NBDs. In addition, the fluorescence of the NBDs is very sensitive to Fe^3+^, which can be used for Fe^3+^ detection in terms of their health superiority. The limit of detection (LOD) of the proposed sensor was determined as 2.9 μM, which is lower than the maximum allowable level of Fe^3+^ (5.37 μM) in drinking water.

**Electronic supplementary material:**

The online version of this article (10.1186/s11671-019-2950-x) contains supplementary material, which is available to authorized users.

## Background

Luminescent nanomaterials have achieved a wide variety of applications due to their unique optical properties, especially in light-emitting diodes, detectors, bioimaging, and metal ion detection [1–6]. Various luminescent nanomaterials have been reported until now, such as semiconductor quantum dots (QDs), carbon nano-dots, and sulfur QDs, which have led to lots of advances in many fields [7–12]. QDs as an excellent representative of luminescent nanomaterials have been used in many fields due to their excellent optical and electrical properties. Despite all this, the toxicity of QDs still limits their applications greatly [13, 14]. It is always of great importance to find greener and more sustainable nanomaterials with luminescence. Biomass is an original organic matter which can be produced via photosynthesis, and it is highlighted for its sustainable and renewable properties. Specifically, biomass is defined as the biodegradable fraction of products, waste, and residues of an organism [15, 16]. In the context of nanotechnology, biomass is usually used as a precursor, and it can be turned into nano-dots with some certain optical properties after special treatment. Compared with chemical precursors, the main components of biomass, especially edible biomass, are sugars and proteins, which are harmless in subsequent treatments. Therefore, the nano-biomass dots (NBDs) should be of high biocompatibility, which ensures their applications in biological and environmental fields without producing harmful substances.

Up to now, only biomass-derived fluorescent carbon nano-dots have been reported. Basically, some natural biomass such as leaves, egg white, and lemon juice were treated by hydrothermal method to synthesize fluorescence carbon nanoparticles [17–19]. There is also another kind of carbon nano-dots that exist in edible foods, which are produced in the further processing of natural biomass [20, 21]. Without exception, all of them involved typical processes of high-temperature carbonization. This process may involve a long time and high temperature, and it is difficult to achieve large-scale batch production [22]. Compared with high temperatures, room temperature or low-temperature conditions are easy to be performed and maintain the original properties of biomass itself.

Nanoprobe is one of the important applications of luminescent nanomaterials [23]. In view of the bright fluorescence and high biocompatibility, NBDs may be used as a kind of nanoprobe in the field of biology and environment. Fe^3+^ is an important metal ion in the human body for which they play a significant role in the synthesis of hemoglobin and myoglobin [24]. But excessive Fe^3+^ accumulation in the body can lead to tissue damage and organ failure. Development of effective and greener sensing systems for qualitative and quantitative determination of Fe^3+^ is of great significance for clinical, medical, and environmental concerns. This allows us to consider whether biomass can be tailored into nano-dots with desirable properties directly from natural edible biomass without any processing. However, none of such luminescent NBDs have been reported to the best of our knowledge. Therefore, looking for more natural biomass precursors to obtain NBDs with desirable properties and high biocompatibility may take a step towards greener luminescent nanomaterials and Fe^3+^ detection.

Herein, luminescent nano-biomass dots (NBDs) have been demonstrated via ultrasonic extraction strategy (UES) from soybeans for the first time. The photoluminescence (PL) quantum yield (QY) of the as-prepared NBDs can reach 16.7%, and the NBDs show bright emission in the solid state. The cytotoxicity test shows that the NBDs have high biocompatibility. Additionally, the NBDs have been employed for Fe^3+^ detection for its fluorescence intensity dependence linearly on the Fe^3+^ concentration, and the limit of detection (LOD) can reach 2.9 μM.

## Methods

### Materials

Varieties of northeast soybeans in line with National Standard of the People’s Republic of China (*GB1352-2009*) were purchased from the local supermarket and washed several times with distilled water before use. Calcium chloride (CaCl_2_), manganese chloride (MnCl_2_), cupric chloride (CuCl_2_), cobaltous chloride (CoCl_2_), lead nitrate (Pb (NO_3_)_2_), and chromium nitrate (Cr(NO_3_)_3_) were purchased from Aladdin Ltd. (Shanghai, China). Ferric chloride (FeCl_3_), ferrous chloride (FeCl_2_), cadmium chloride (CdCl_2_), mercury dichloride (HgCl_2_), sodium chloride (NaCl), and zinc chloride (ZnCl_2_) were obtained from Sinopharm Chemical Reagent Co., Ltd. (Shanghai, China). All chemicals are of analytical reagent (purity > 99.0%) and used as received without further purification.

### Synthesis of NBDs

Firstly, 100 pieces of soybeans were washed with a mixture of alcohol and distilled water for 3 times to remove the impurity. Then the soybeans were placed into a beaker with 50 ml distilled water followed by ultrasonic for 2 h. During this process, the color of the solution changed from transparent to dark yellow, indicating the peel of soybean was tailored into nano-size to form NBDs. Then, the dark yellow solution was transferred into centrifuge tubes and centrifuged at 7000 rpm for 3 mins twice to remove large-size particles, after that the supernatant was filtered through a 0.22-μM membrane to remove large or agglomerated particles further. Whereafter, the solution was placed in a refrigerator followed by frozen treatment at − 5 ^°^C for 6 h. Then, it was transferred to a lyophilizer at − 50 ^°^C for 12 h to obtain the powders. The frozen powders were dispersed into water to form NBDs for further application.

### Characterization

The X-ray diffraction (XRD) pattern of the NBDs was recorded using an X′ Pert Pro diffractometer, in which X-rays were generated by a Cu-Kα source. A JEM-2010 transmission electron microscope (TEM) was employed to characterize the size and crystallinity of the NBDs. The fluorescence spectra of the NBDs were obtained with an F-7000 fluorescence spectrophotometer. The UV-Vis absorption spectra of the NBDs were obtained using a UH4150 spectrophotometer. The fourier-transform infrared (FTIR) spectra of the samples were recorded by a Thermo Scientific Nicolet iS10 FTIR spectrometer. The X-ray photoelectron spectroscopy (XPS) spectra of the samples were collected by using a Thermo Fisher Scientific ESCALAB 250Xi spectrometer equipped with an Al-Kα X-ray radiation source.

### Photoluminescence Quantum Yield Measurement

The PL QY was tested using an F-9000 spectrofluorometer with integrating sphere. First of all, the NBD aqueous solution was diluted to an absorption intensity of below 0.1. Then, this aqueous solution was added into a fluorescence cuvette, placed in the integrating sphere, and excited with 370-nm monochromatic light. The fluorescence spectra were collected in the ranges of 430–450 nm. Meanwhile, the same fluorescence spectra for pure water were also recorded under identical conditions. Finally, the PL QY was calculated using fluorescence software based on the PL spectra of both the sample and the water.

### Cellular Toxicity Test

The cytotoxicity of NBDs is evaluated by MTT (3-(4,5)-dimethylthiahiazo(-z-y1)-3,5-di-phenytetra-zoliu-mromide) methods. Cells were cultured in normal RPMI-1640 with 10% fetal bovine serum in 5% CO_2_ and 95% air at 37 ^°^C in a humidified incubator. For cell viability measurements, HeLa cells were placed in 96-well plates and then incubated for 72 h. After the incubation of Hela cells with various concentrations of NBDs and CDs for 72 h, the cell viability was recorded.

### Detection of Fe^3+^

A 1 ml of solution with different concentrations of Fe^3+^ was added into 1 ml of NBDs with 3 g/l solution before the PL measurements. The solutions were mixed thoroughly and left to react for 1 min at room temperature, and then recorded the associated fluorescence spectra. The PL measurements were performed under excitation of 370 nm.

## Results and Discussion

### Morphology and Chemical Composition

**Scheme 1:**
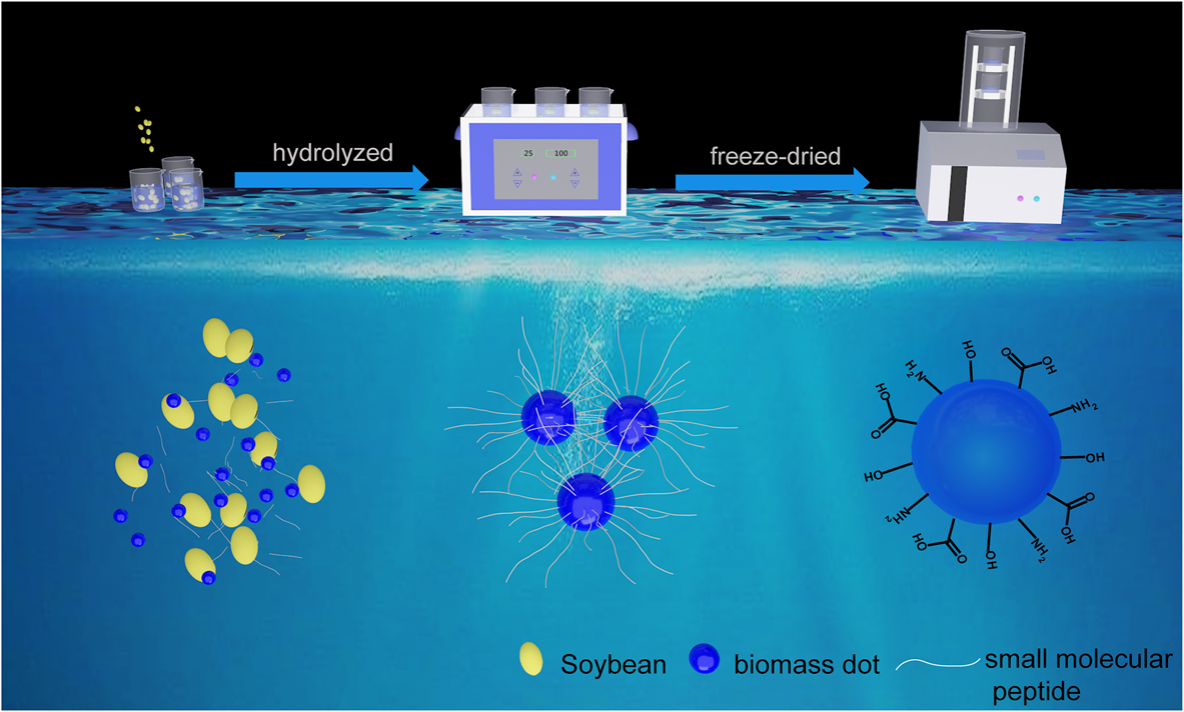
Schematic illustration of the preparation process of the NBDs from soybeans

**Fig. 1:**
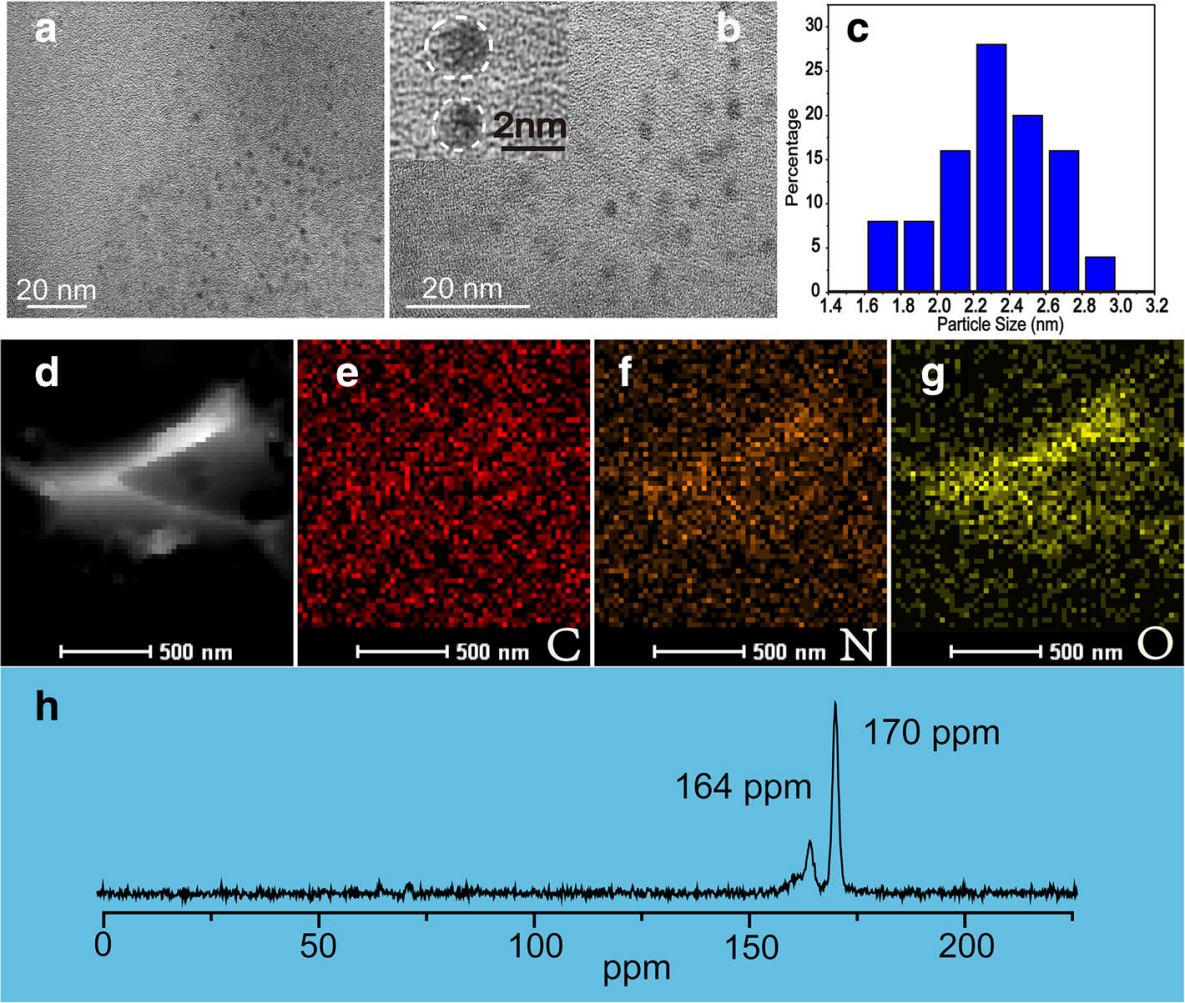
TEM images of the NBDs (**a**) and (**b**). **c** The particle size distribution of the NBDs. HAADF image (**d**) and corresponding elemental distribution mapping of carbon (**e**), nitrogen (**f**), and oxygen (**g**)

The NBDs have been prepared via UES methods; all of the processes are illustrated in Scheme 1. The sizes and morphology of NBDs were characterized by transmission electron microscopy (TEM), as shown in Fig. 1a and b. The TEM images show that the NBDs were nearly spherical in shape. The diameters of the NBDs are ranged from 1 to 3 nm with an average diameter of 2.4 nm, and the corresponding size distribution is listed in Fig. 1c. Lattice fringes of the NBDs cannot be observed from the high-resolution TEM image (inset of Fig. 1b), indicating the amorphous nature of the NBDs. The images of the high-angle annular dark-field scanning transmission electron microscopy (HAADF-STEM) and the corresponding elemental mapping (carbon, nitrogen, and oxygen) of the NBDs are shown in Fig. 1d–g. It can be seen that the dominant elements of the NBDs are carbon, nitrogen, and oxygen. Furthermore, solid-state ^13^C nuclear magnetic resonance (NMR) measurements of the NBDs are shown in Fig. 1h. The signals are ranged from 160–180 ppm, and the peaks at 164 ppm and 170 ppm are corresponding to C=O bonds, which are indicative of sp^2^ carbon atoms [25, 26].

**Fig. 2:**
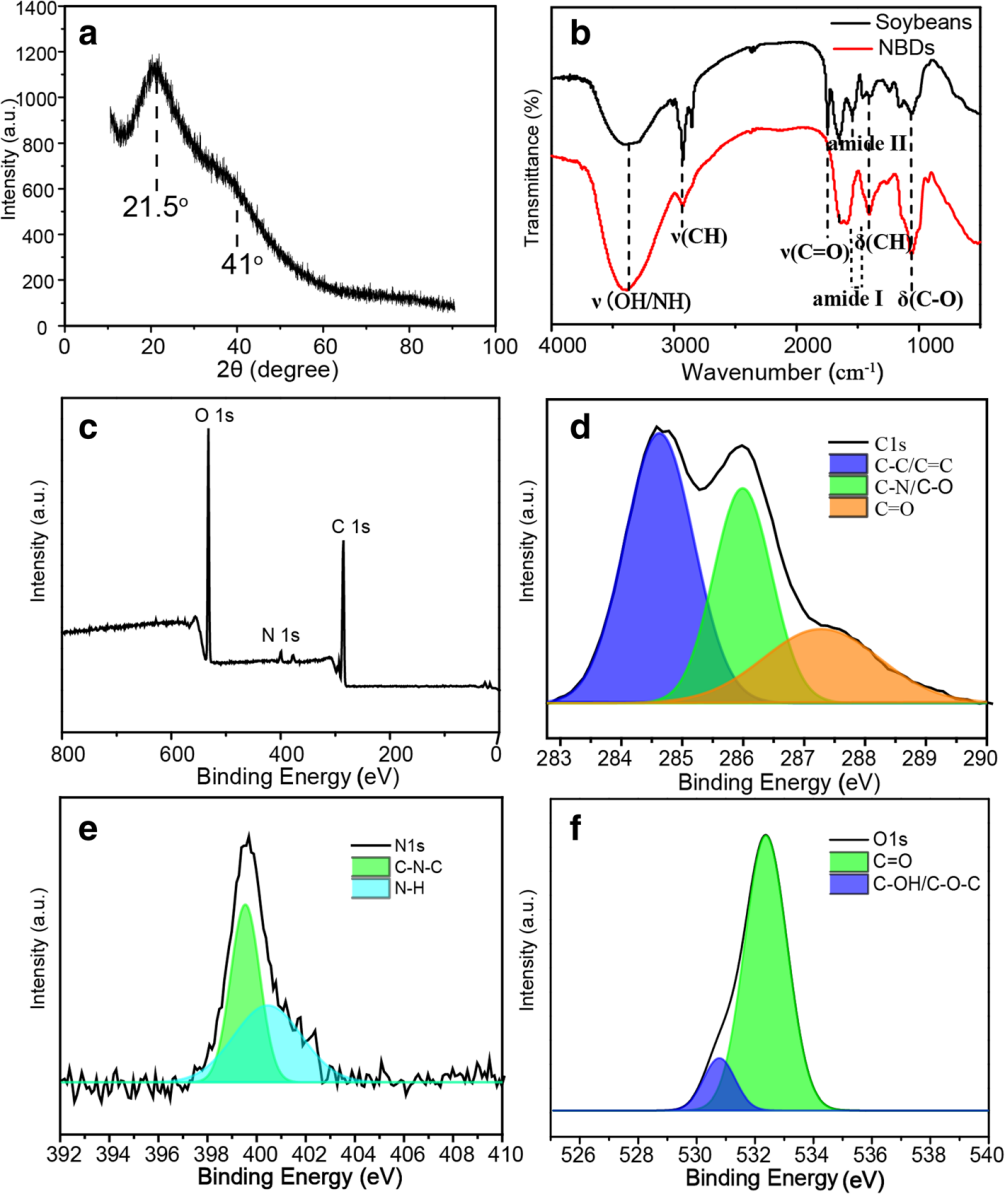
**a** XRD pattern of the NBDs. **b** FTIR spectra of the soybean and NBDs. **c** XPS survey spectrum of the NBDs. High-resolution XPS spectra of C 1s (**d**), N 1s (**e**), and O 1s (**f**)

To further study the structural characteristics of the NBDs, X-ray diffraction (XRD) pattern was recorded. As shown in Fig. 2a, the typical XRD pattern displays a broad peak located at around 21.5^o^ and a shoulder peak at around 41.0^o^, which can be attributed to the amorphous carbon phase [27]. Furthermore, the characteristic absorption peaks of the soybeans and NBDs were investigated by fourier-transform infrared (FTIR) spectroscopy, as shown in Fig. 2(b). The absorption bands at around 3380 cm^−1^ can be assigned to the stretching vibrations of O–H/–N–H, the band at around 2906 cm^−1^ to the C–H stretching vibrations, and the band at around 1650 cm^−1^ to the C=O stretching vibrations. The peaks at 1400 cm^−1^ and 1071 cm^−1^ are corresponding to C–H and C–O bending vibrations, respectively [28]. There are an obvious difference between the spectrum of soybeans and NBDs at around 1750 cm^−1^, which belongs to the stretching vibrations of C=O bonds from the lipids in soybeans [29, 30]. The insoluble lipids in aqueous solution were separated from the sample when they are soaking in water, leading to the disappearance of the bonds in the FTIR spectrum of the NBDs. The reduced C=O bonds in the sample come from the carboxyl group in the protein. The peak centered at about 1543 cm^−1^ was also disappeared, which can be attributed to the proteolysis in the soaking process of the soybeans. While comparing all peaks before and after the ultrasound process, the formation of –OH, –C=O (amide I), and –NH groups on the surface of the NBDs can be seen [31]. The above results demonstrate the existence of hydroxyl, amidogen, and carboxylic groups on the surface of the NBDs, and these functional groups play an important role in the hydrophilicity and stability of the NBDs in aqueous solution. X-ray photoelectron spectroscopy (XPS) spectra were performed to further elucidate the components of the NBDs, as shown in Fig. 2c. The XPS spectrum shows three strong peaks at 532.0, 401.1, and 286.1 eV, which can be attributed to O 1s, N 1s (Fig. 2d), and C 1s (Fig. 2e), respectively [32]. These results indicate that the NBDs mainly contain C (64.33%), O (32.34%), and N (2.72%), as well as limited amount of P, and the P element may come from the phospholipid of the soybeans [33]. In the high-resolution XPS spectrum, the C 1s spectrum displays three peaks at 287.6, 285.8, and 284.6 eV, which can be assigned to the C=O, C–O/C–N, and C–C/C=C groups, as shown in Fig. 2c. The C=O bond is from the soluble carboxyl groups [24]. The C–O/C=N and C–C/C=C are from the nitrous carbons and sp^2^/sp^3^ carbons, respectively [34]. The N 1s spectrum shown in Fig. 2d confirms two main bands at 399.5 eV and 401.6 eV, revealing the existence of pyridinic N and pyrrolic N, which is consistent with the FTIR analysis. The O 1s spectrum presented in Fig. 2f has two peaks at 531.4 eV and 533.0 eV, which can be attributed to the C–OH/C–O–C and C=O groups, respectively [9].

A possible mechanism for the formation of NBDs from soybeans was proposed based on the above analysis. First, some large particles of biomass suspended in the solution are broken into nanometer size by the ultrasonic concussion. Changes in solution before and after ultrasonic extraction treatment are shown in Additional file 1: Figure S1. Then, the protein in the soybeans was hydrolyzed to small molecular peptide and amino acid in the above process, and a lot of small molecule peptide chains are attached to the nano-size biomass to form the highly surface functionalized biomass dots. The functional groups on the surface of the biomass dots are the major contributors to fluorescence. According to the mechanism, the mung bean was also used as precursors, and blue fluorescent NBDs were also obtained, as shown in Additional file 1: Figure S2.

### Optical Properties

**Fig. 3:**
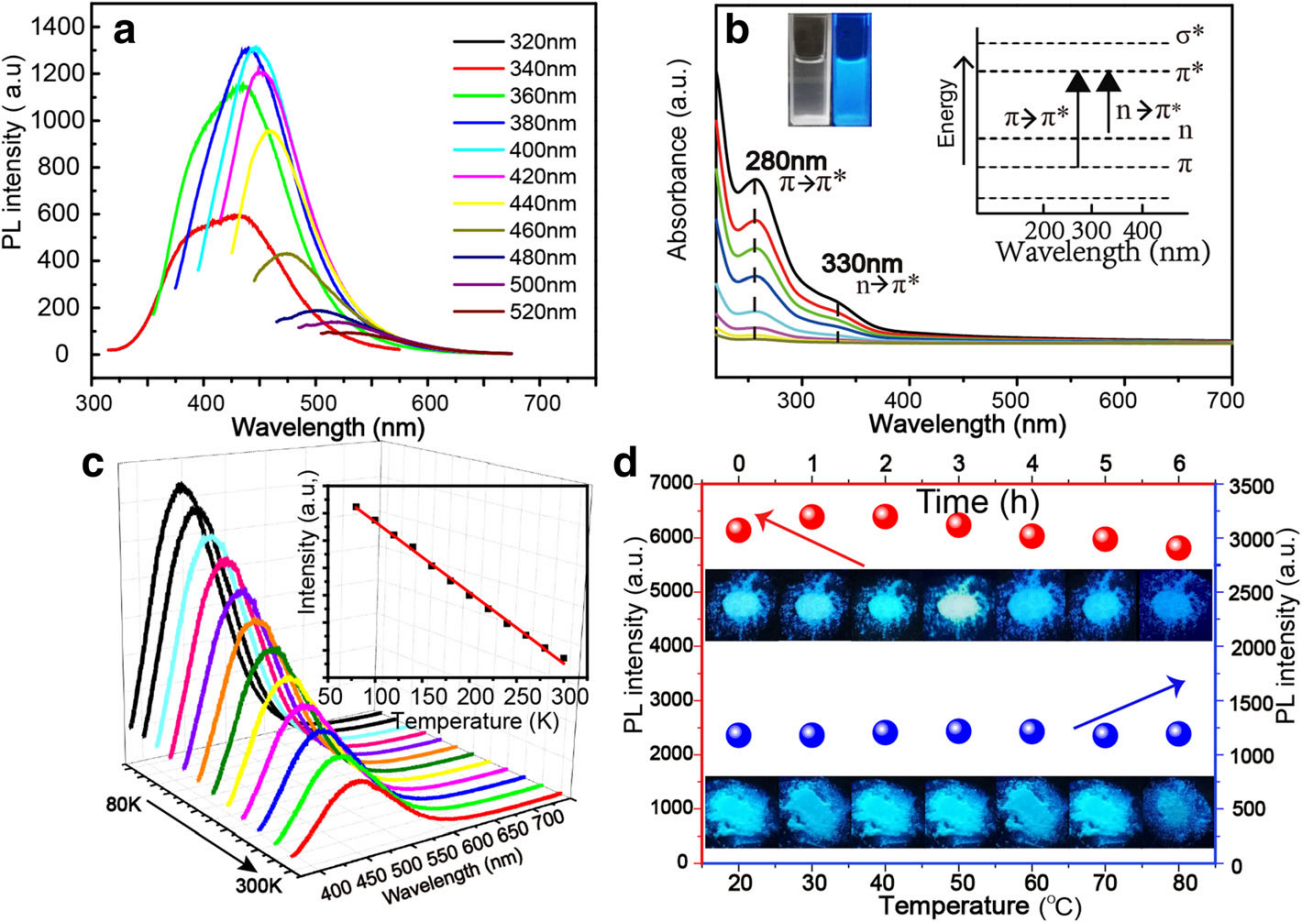
**a** Fluorescence spectra of the NBDs with excitation wavelength changes from 320 to 520 nm. **b** UV-Vis absorption spectra of the NBDs. **c** Fluorescence spectra of the NBDs at different temperature, the inset is the plot of the fluorescence intensity of the NBDs as a function of temperature. **d** Fluorescence intensity and images of the NBD powders under the illumination of a 365-nm lamp for different duration and those of the NBD powders at different measuring temperature

The NBDs show excitation-dependent fluorescent properties, and when the excitation wavelength varies from 320 to 520 nm, the emission peak redshifts gradually, indicating that the emission of the NBDs can be tunned by changing the excitation wavelength, as shown in Fig. 3a. The NBD aqueous solution is transparent under indoor lighting and shows blue fluorescence under UV illumination, as shown in the inset of Fig. 3b. The photoluminescence excitation (PLE) spectrum of the NBDs is shown in Additional file 1: Figure S3, and the optical excitation wavelength is in the range of 360 to 420 nm. To explore the PL origin of the NBDs, the UV-Vis absorption spectra of the NBDs with different concentrations have been recorded at room temperature (the concentration of NBDs from bottom to top is 0.03, 0.06, 0.13, 0.25, 0.25, 0.50, 0.50, 0.75, 1.00, and 1.50 g/l), as shown in Fig. 3b. The UV–Vis absorption spectra of the NBDs exhibit two clear absorption peaks at 270 nm and 330 nm, respectively. The former can be attributed to the π-π^*^ transition of C–C/C=C bonds, while the latter to the n-π^*^ transition of C=O/N bonds [35, 36]. These functional groups are the major chromogenic groups that contribute to the fluorescence of the NBDs [37, 38]. The PL spectra of the soybeans during ultrasonic extraction are shown in Additional file 1: Figure S4, and the PL intensity increases with time then reaches the maximum. Figure 3c shows the PL spectra of the NBDs measured from 80 to 300 K. The NBDs exhibit a typical thermal quenching behavior, in which all peaks decrease monotonically in intensity with increasing temperature. This PL behavior can be attributed to the increase of non-radiative recombination and the reduction of radiation recombination with the increase of temperatures [39, 40]. To evaluate the stability of the NBDs, the photostability and thermostability of the NBDs have been characterized, as shown in Fig. 3d. For photostability, the measurement setup image is shown in Additional file 1: Figure S5. The fluorescence intensity values are shown in Additional file 1: Figure S6 and S7. The emission intensity of the NBDs remains above 90% under the UV lamp illumination for 6 h, indicating their good photostability. For thermostability, the fluorescence intensities of the NBDs decrease little when the temperature varied from 20 to 80 ^°^C, revealing their high thermal stability.

### Cytotoxicity Evaluation

**Fig. 4:**
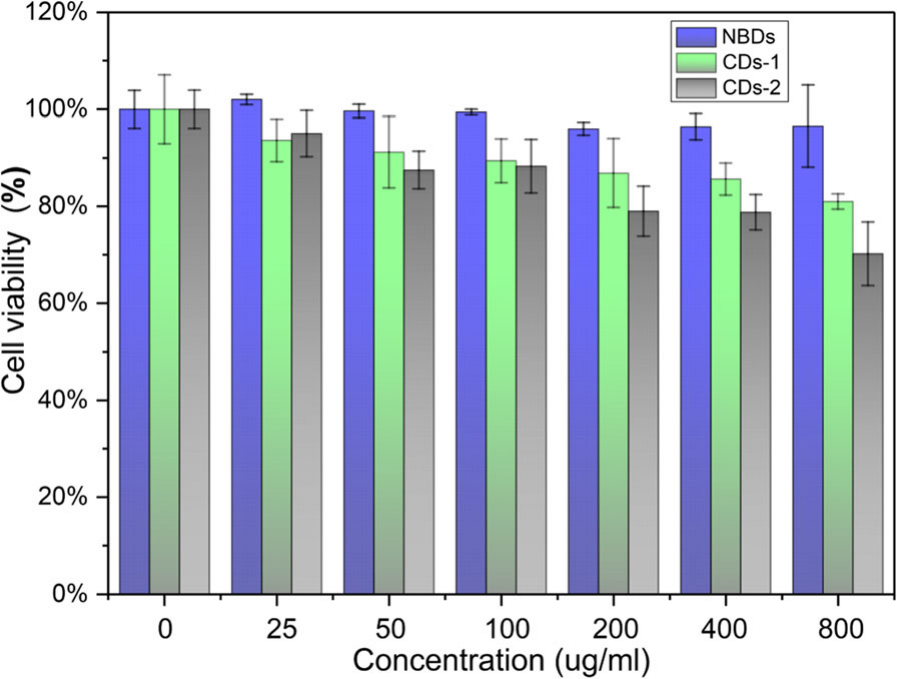
Viability of HeLa cells after 72 h of incubation with different concentrations of NBDs and CDs

The MTT assays were used to evaluate the cytotoxicity of the NBDs. The viabilities of HeLa cell incubated with the NBDs and two other kinds of CDs synthesized via hydrothermal method, as shown in Fig. 4. As indicated in the figure, the cell viabilities decrease little when the NBD solution is introduced even when the concentration of the NBDs reaches 800 μg/ml. The cell survival rate was 70% and 67% when the HeLa cells were incubated with the other two kinds of CDs at a concentration of 800 μg/ml. Obviously, the NBDs exhibit superior biocompatibility than the CDs prepared from chemical reagents.

### Sensing Properties of the NBDs Toward Fe^3+^

**Fig. 5:**
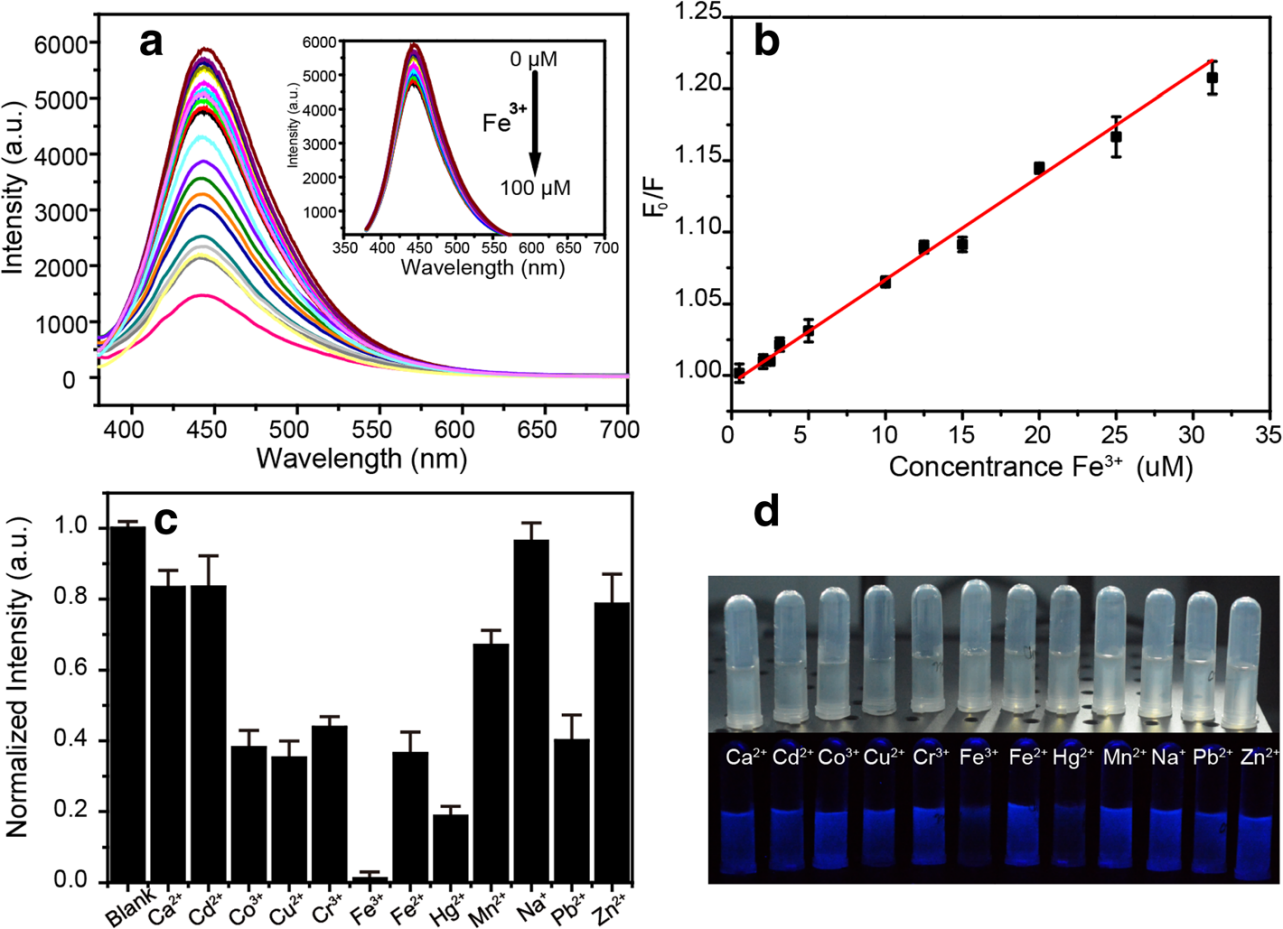
**a** PL spectra of the NBDs in the presence of different concentrations of Fe^**3**+^. **b** Calibration curve of the sensor as a function of Fe^**3**+^ concentration. **c** Fluorescence intensities of the NBDs in the presence of different ions. **d** Photographic images of the NBDs solution with different metal ions under indoor and UV illumination

Interestingly, the fluorescence of the NBDs can be effectively quenched by Fe^3+^, as shown in Fig. 5a, and the PL intensity of the NBDs decreases significantly with the increase of Fe^3+^ concentration. Moreover, a good linear relationship can be plotted between F_0_/F and the Fe^3+^ concentration ranging from 0 to 30 μM (*R*^2^ = 0.99), where F_0_ and F were the PL intensity of the NBDs at ex/em of 370/445 nm in the absence and presence of Fe^3+^, as shown in Fig. 5b. The quenching efficiency was fitted by the Stern-Volmer Eq:

1 $$ \frac{{\mathrm{F}}_0}{\mathrm{F}}=1+{K}_{\mathrm{SV}}\left[Q\right] $$

where *K*_sv_ is the Stern-Volmer quenching constant and [*Q*] is the Fe^3+^ concentration. The linear regression equation is *Y* = 0.0072*X* + 0.99479, *R*^2^ = 0.99. The limit of detection (LOD) of the proposed sensor was determined as 2.9 μM, which is lower than the maximum allowable level of Fe^3+^ (5.37 μM) in drinking water set up by the United States Environmental Protection Agency (USEPA) [24]. Selectivity is another critical parameter for chemical sensors. Hence, the fluorescence response of the sensor towards several interfering metal ions has been investigated, including Ca^2+^, Cd^2+^, Co^2+^, Cu^2+^, Cr^3+^, Fe^3+^, Fe^2+^, Hg^2+^, Mn^2+^, Na^+^, Pb^2+^, and Zn^2+^. Metal ions each at a concentration of 10^−2^ M were added into 1 ml of NBDs solution with a concentration of 3 g/l. In Fig. 5c, it can be seen that the fluorescence intensity of the NBDs responds more sensitively to Fe^3+^ than other metal ions. The photographs in Fig. 5d are the images of the NBDs with various ions under indoor and UV illumination, and the concentration of metal ions was 100 μM. Obviously, the NBDs are quenching in the presence of Fe^3+^, indicating they can be used for visual detection.

### Quenching Mechanism

**Fig. 6:**
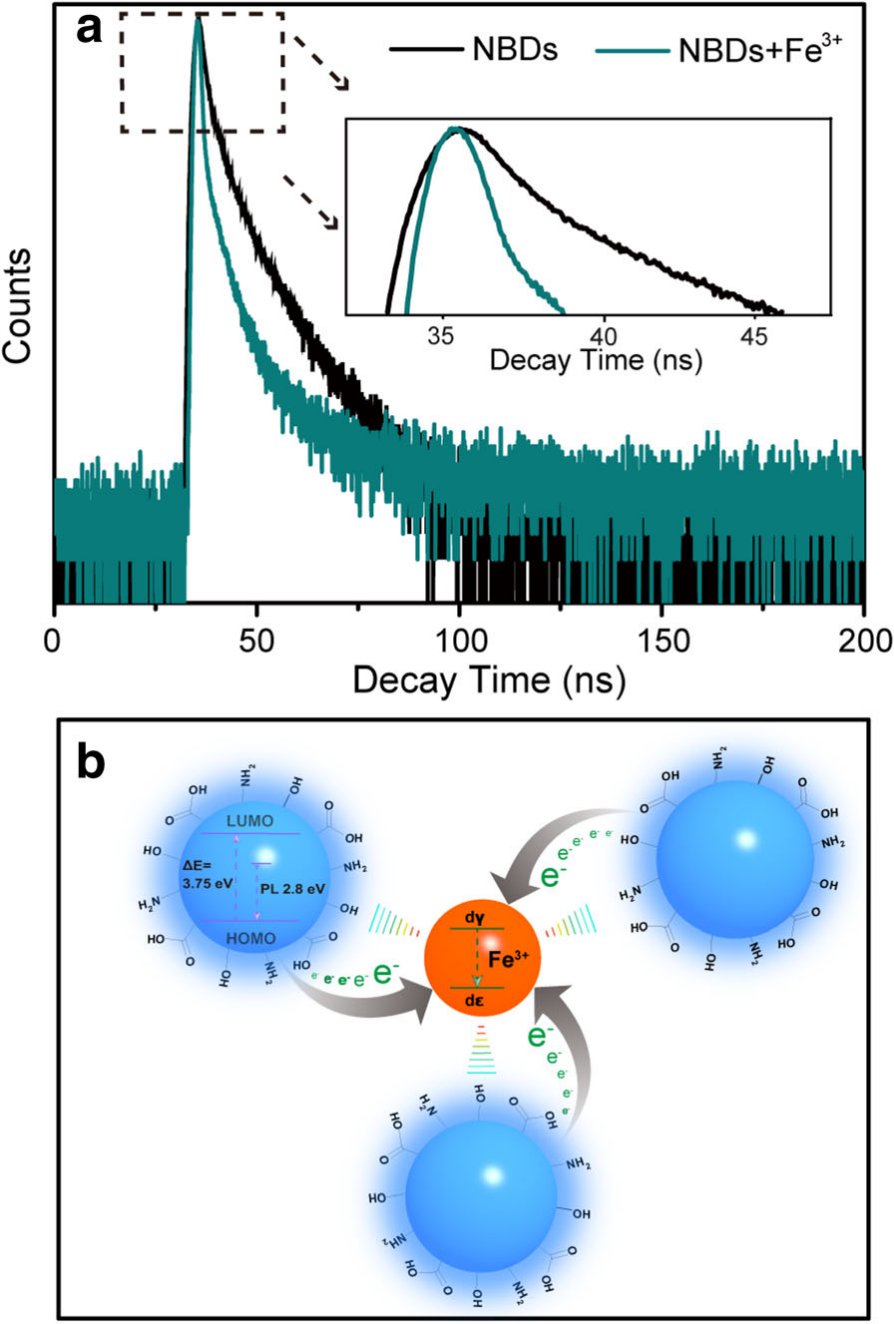
**a** Fluorescence decay traces of the NCDs in the absence and presence of Fe^**3**+^ under excitation at 370 nm and emission at 445 nm. **b** Schematic illustration for the possible fluorescence quenching mechanism of the NBDs in the presence of Fe^**3**+^ ions

The quenching mechanism of the NBDs in the presence of Fe^3+^ was discussed based on the UV-Vis absorption spectra and fluorescence lifetime of the NBDs. From the UV-Vis absorption spectra shown in Additional file 1: Figure S8, there is no change of the absorption peaks at 270 nm and 340 nm with the introduction of Fe^3+^, indicating that the Fe^3+^ does not influence the structure of the NBDs [41]. Apart from the UV-Vis absorption spectrum, the effect of Fe^3+^ on the lifetime of the NBD was also studied. In Fig. 6a, the fluorescence lifetime becomes shorter after the addition of Fe^3+^, which may involve partial electron transfer of the NBDs to the *d* orbital of Fe^3+^, thus decrease the radiative recombination of the NBDs [42]. The fluorescence quenching mechanism of the NBDs caused by Fe^3+^ is shown in Fig. 6b. The sensitive fluorescence quenching effect of the NBDs in the presence of Fe^3+^ may originate from the strong interaction between Fe^3+^ and the surface groups of the NBDs. Fe^3+^ has a stronger binding affinity and faster chelating kinetics with amino and carboxylic groups on the surfaces of NBDs. The special coordination between the Fe^3+^ ions and the phenolic hydroxyl/amine groups of NBDs has been widely used for the detection of Fe^3+^ ions or colored reactions in traditional organic chemistry [43, 44]. In addition, the redox potentials of Fe^3+^/Fe^2+^ (*Ф* = 0.77) are located between the lowest unoccupied molecular orbital (LUMO) and highest occupied molecular orbital (HOMO) of the NBDs, causing photo-induced electron transfer from LUMO to the complex states of Fe^3+^ [45]. These results demonstrate that the NBDs are highly sensitive to Fe^3+^ over the other metal ions.

## Conclusion

In summary, luminescent NBDs have been prepared from soybean via a heating-free UES approach. The NBDs show bright blue fluorescence with PL QY of 16.7%, and benefiting from the edible biomass and heating-free synthesis process, the cell viability still keeps 100% even if the concentration of the NBDs reaches 800 μg/ml. In addition, the fluorescence of the NBDs exhibits specific sensitivity to Fe^3+^, and the LOD can reach 2.9 μM. The low toxicity and high detection limit indicate that the NBDs are expected to find potential applications in biological and environmental systems.

## Additional file

Additional file 1: Figure S1. Change of soybean solution before and after ultrasonic extraction treatment. Figure S2. (a) UV-Vis absorption and PL spectra of the NBDs by UES from mung bean. (b) XRD pattern. (c) FTIR spectrum. (d) XPS of the NBDs. Figure S3. Fluorescence and excitation spectra of the as-prepared NBDs. Figure S4. The PL spectra of the soybeans during ultrasonic extraction. Figure S5. The images of the NBD powders under UV illumination (0.15 mW/cm2) and corresponding testing process. Figure S6. The photostability of the NBDs after irradiation for 6 h. Figure S7. The thermostability of the NBDs at different temperatures. Figure S8. Absorption spectra of the NBDs with Fe^3+^ and without Fe^3+^. (DOCX 1231 kb)

## Abbreviations

FTIR: Fourier-transform infrared
HAADF-STEM: High-angle annular dark-field scanning transmission electron microscopy
LOD: Limit of detection
NBDs: Nano-biomass dots
NMR: Nuclear magnetic resonance
PL: Photoluminescence
QDs: Quantum dots
QY: Quantum yield
TEM: Transmission electron microscopy
UES: Ultrasonic extraction strategy
USEPA: United States Environmental Protection Agency
XPS: X-ray photoelectron spectroscopy
XRD: X-ray diffraction
