# Supplementary material for: Fluorescent Nano-Biomass Dots: Ultrasonic-Assisted Extraction and Their Application as Nanoprobe for Fe3+ detection
Source: Nanoscale Res Lett. 2019 Apr 15;14:130. doi: 10.1186/s11671-019-2950-x (PMC6465388; doi:10.1186/s11671-019-2950-x)
Supplement: Supplementary file 1 — Figure S1. Change of soybean solution before and after ultrasonic extraction treatment. Figure S2. (a) UV-Vis absorption and PL spectra of the NBDs by UES from mung bean. (b) XRD pattern. (c) FTIR spectrum. (d) XPS of the NBDs. Figure S3. Fluorescence and excitation spectra of the as-prepared NBDs. Figure S4. The PL spectra of the soybeans during ultrasonic extraction. Figure S5. The images of the NBD powders under UV illumination (0.15 mW/cm2) and corresponding testing process. Figure S6. The photostability of the NBDs after irradiation for 6 h. Figure S7. The thermostability of the NBDs at different temperatures. Figure S8. Absorption spectra of the NBDs with Fe3+ and without Fe3+. (DOCX 1231 kb) [file 11671_2019_2950_MOESM1_ESM.docx]

**Fluorescent Nano-Biomass Dots: Ultrasonic-Assisted Extraction and Their Application as Nanoprobe for Fe^3+^ detection**

Wen-Bo Zhao, Kai-Kai Liu, * Shi-Yu Song, Rui Zhou, and Chong-Xin Shan*

*Henan Key Laboratory of Diamond Optoelectronic Materials and Devices, School of Physics and Engineering, Zhengzhou University, Zhengzhou, 450052, People’s Republic of China.*


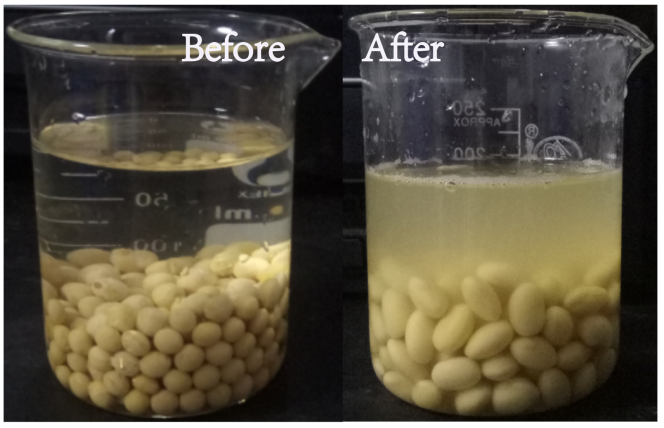


**Figure S1**. Change of soybean solution before and after ultrasonic extraction treatment.


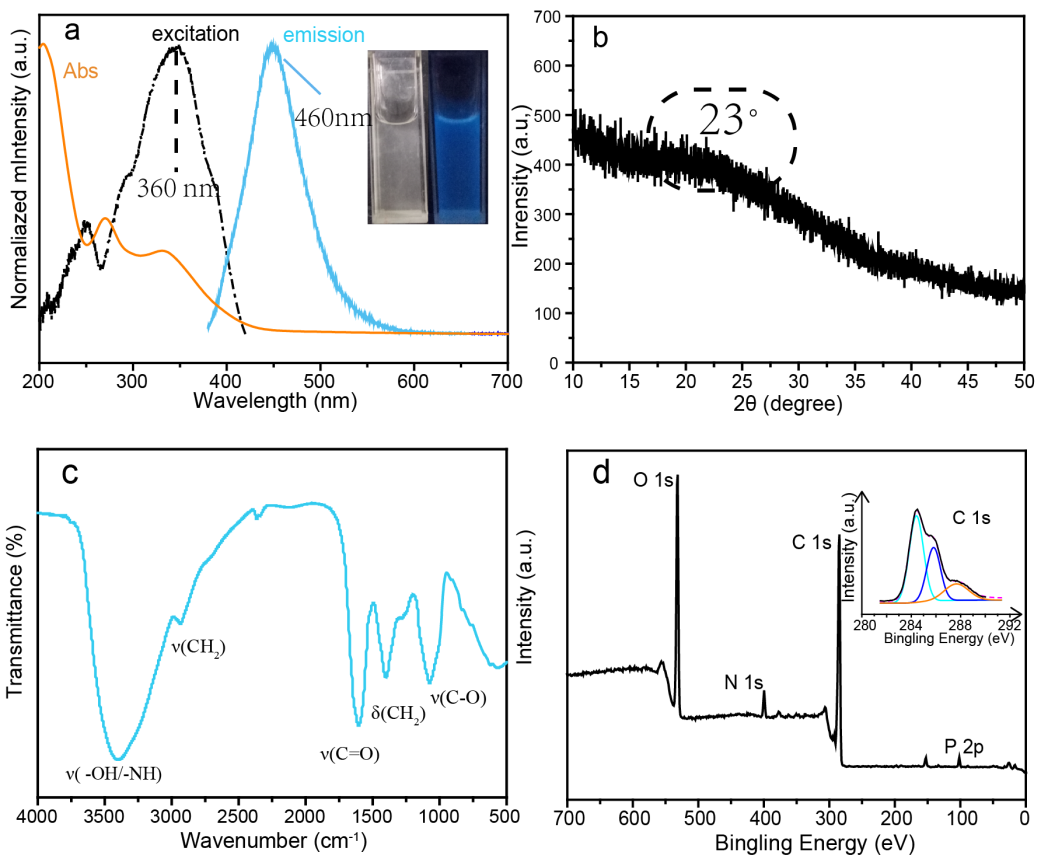


**Figure S2.** (a) UV-vis absorption and PL spectra of the NBDs by UES from mungbean. (b) XRD pattern. (c) FTIR spectrum and (d) XPS of the NBDs.


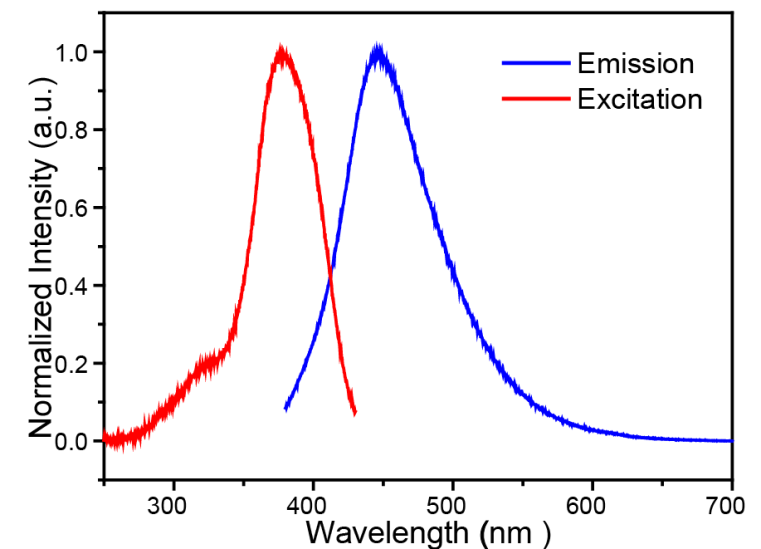


**Figure S3.** Fluorescence and excitation spectra of the as-prepared NBDs.


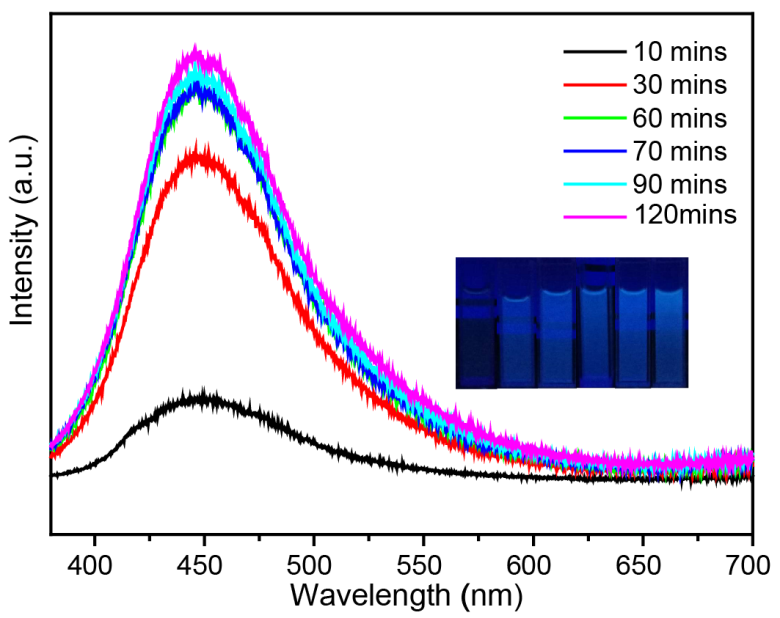


**Figure S4.** The PL spectra of the soybeans during ultrasonic extraction.


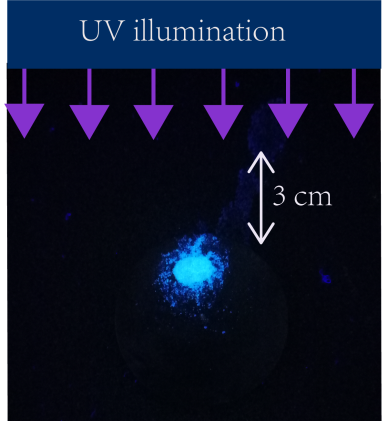


**Figure S5.** The images of the NBDs powders under UV illumination (0.15 mW/cm^2^) and corresponding testing process.


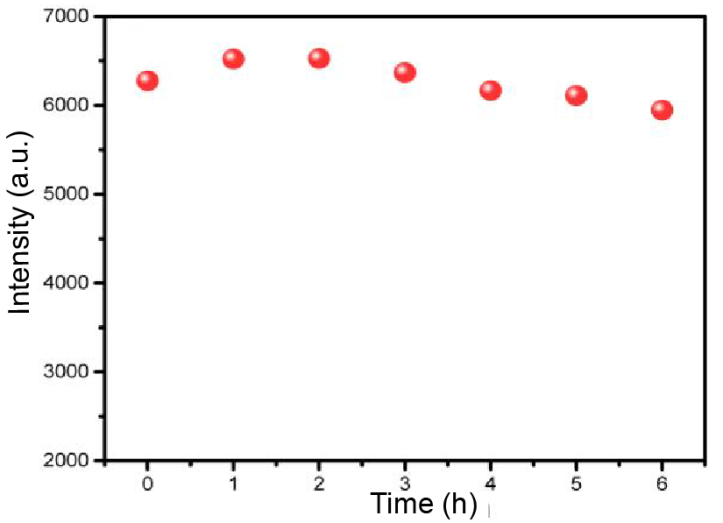


**Figure S6.** The Photo-stability of the NBDs after irradiation for 6 hours.


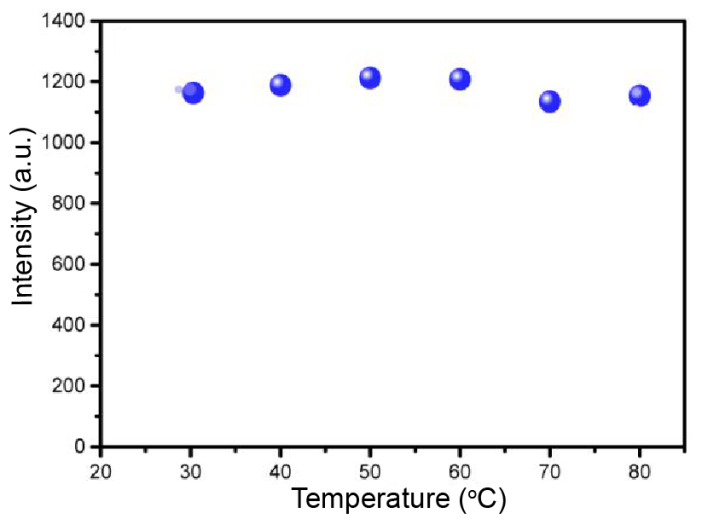


**Figure S7.** The thermo-stability of the NBDs at different temperatures.


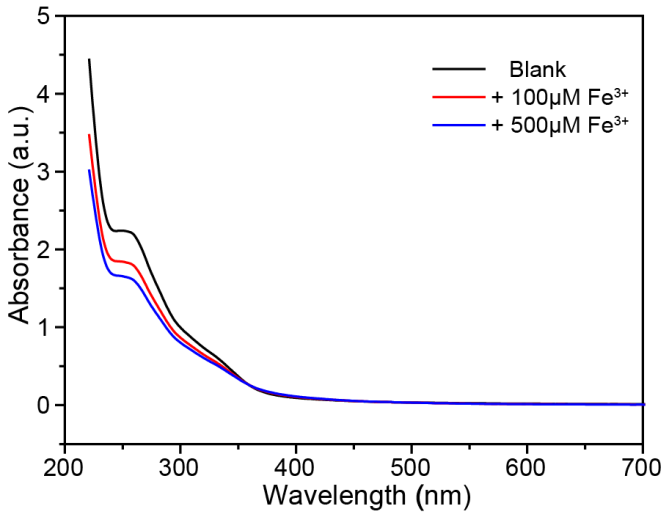


**Figure S8.** Absorption spectra of the NBDs with Fe^3+^ and without Fe^3+^.
